# Discovery of novel antifungal drugs via screening repurposing libraries against *Coccidioides posadasii* spherule initials

**DOI:** 10.1128/mbio.00205-25

**Published:** 2025-03-26

**Authors:** Sarah Saeger, Kathryn West-Jeppson, Yu-Rou Liao, Althea Campuzano, Jieh-Juen Yu, Jose Lopez-Ribot, Chiung-Yu Hung

**Affiliations:** 1 Department of Molecular Microbiology and Immunology, South Texas Center for Emerging Infectious Diseases, The University of Texas at San Antonio 12346 , San Antonio, Texas, USA; University of Toronto, Toronto, Ontario, Canada

**Keywords:** coccidioidomycosis, valley fever, drug repurposing, antifungal drug discovery, assay development, *Coccidioides*, spherules, high-throughput screening

## Abstract

Coccidioidomycosis or valley fever is a treatment-limited fungal infection endemic to the alkaline deserts of North and South America for which two classes of antifungals are typically used: the polyenes and the triazoles. In light of the limited usefulness of the echinocandins and a growing trend of azole resistance, it is essential that we identify novel antifungals. In this study, we have developed and optimized a screening methodology for identifying potential antifungals effective against *Coccidioides* spherule initials using a metabolic assay, used it to screen four diverse drug libraries with limited drug overlap, and established safety and efficacy data for a majority of the compounds, including the Broad Repurposing Hub, Prestwick Chemicals 1520, Selleck L8200 Anti-parasitic, and MedChemExpress CNS Penetrants libraries. Hits were defined as compounds with strong metabolic inhibition (≥70%), which were significantly different compared to the median plate readout (B-scores ≤ −3). We identified 30 promising hits and found 12 compounds exhibiting half-maximal inhibitory concentrations below 6 µM. Among these, oxethazaine, niclosamide ethanolamine, 10058-F4, niclosamide (NIC), and pentamidine isethionate showed synergy with amphotericin B, suggesting their potential use in combination therapy. Further assessment of lead compounds’ effects on spherules was conducted by image flow cytometry. Additionally, we explored the potential to use an attenuated, Biosafety Level 2 containment mutant, *C. posadasii* ∆*cts2*/∆*ard1*/∆*cts3* (∆T), as a surrogate model for drug screening. Overall, our findings provide a foundation for future research focused on screening and developing novel coccidioidomycosis treatments.

**IMPORTANCE:**

The antifungal treatment arsenal is especially limited against *Coccidioides*. Due to toxicity concerns, amphotericin B is generally reserved for triazole-recalcitrant infections. Recent laboratory susceptibility tests show an increase in fluconazole resistance, highlighting a need for new treatments. We have developed a large-scale metabolic screening assay under Biosafety Level 3 containment to identify existing drugs with novel activity against *Coccidioides* spherules. This drug-repurposing approach represents a convenient and cost-effective strategy to increase the available antifungals effective against these infections.

## INTRODUCTION

Coccidioidomycosis or valley fever is an emerging mycosis caused by the dimorphic pathogenic fungi *Coccidioides immitis* and *Coccidioides posadasii*, affecting both healthy and immunocompromised individuals in semi-arid American deserts (1, 2). Climate change-induced desertification is expected to expand the range of these pathogens from the Sonoran and Mojave deserts northward toward Wyoming and the Dakotas (3–5). Individuals are exposed to arthroconidia (spores) when contaminated soil is mechanically disturbed, aerosolizing arthroconidia from mycelial filaments. Inhaled arthroconidia undergo significant metabolic and cellular remodeling, swelling isotropically and developing into unique structures, called spherule initials (6). As the spherules mature, they segment, forming internal cellular compartments divided by septa. Each internal cross-walled compartment subsequently differentiates and develops into an endospore. Approximately 5 to 7 days post-inoculation, the mature spherule ruptures, releasing hundreds of endospores, which initiate a subsequent parasitic cycle (6). While mature spherules are extracellular, the smaller endospores (3–7 µm in diameter) may be phagocytosed by immune cells, whereby they disseminate to extrapulmonary tissue/organs via lymphatic or hematogenous routes.

Most exposed individuals are asymptomatic; however, 40% of infections experience flu-like symptoms often misdiagnosed (17–29%) as community-acquired pneumonia, delaying proper treatment (7). Approximately 5–10% of individuals develop pulmonary sequelae, including calcified nodules, granulomas, and lung cavitation, and 1–7% experience progressive dissemination from the lungs to the bone marrow (osteomyelitis), joints (desert rheumatism), skin (cutaneous coccidioidomycosis), and the brain (meningitis). In the latter case, treatment is challenging, and mortality rates can exceed 50% (8). Treatment of systemic coccidioidomycosis is lengthy, costly, and may be needed prophylactically for life, as acquired immunosuppression can revitalize latent infections in granulomas (9–12).

Treatment of systemic fungal infections is limited to just 11 antifungal drugs across four classes: azoles, polyenes, echinocandins, and nucleotide chain terminators (13). Mechanisms of action (MOA) of those drugs target a handful of biological functions, including the cell wall (e.g., caspofungin [CAS], anidulafungin, micafungin), RNA synthesis (e.g., flucytosine), and fungal cell membrane synthesis and integrity (e.g., fluconazole [FLU] and amphotericin B [AmB]). Due to the limited treatment efficacy of echinocandins and flucytosine (8, 14), the treatment of uncomplicated and progressive coccidioidomycosis is primarily by triazoles and AmB, respectively (15). The triazole FLU penetrates the CNS and is generally considered safe for long-term use; however, a recent survey of clinical isolates noted increasing intrinsic FLU resistance (16). AmB, while effective, is generally reserved for severe disseminated infections due to its nephrotoxic effects (17). Thus, the need for novel anti-*Coccidioides* drugs is urgent due to a limited arsenal of clinically available drugs, the risk of antifungal resistance, the high lifetime cost burden, and the rapidly increasing incidence of coccidioidomycosis.

*Coccidioides* is designated as a Biosafety Level 3 (BSL-3) pathogen due to the risk of lethal infection by aerosolized spores; consequently, growing spherules requires specialized containment, techniques, equipment, and facilities. As a result of these challenges, current clinical drug susceptibility testing for *Coccidioides* has only been performed in saprobic arthroconidia mostly following the CLSI M38 protocol (15). Correlations of drug susceptibility among hyphae, arthroconidia, spherules, and endospores morphologies are not known. This study aims to identify compounds inhibiting the early stage of parasitic growth by targeting clinically relevant spherule initials.

Two approaches have been historically applied to develop novel antifungals and innovative treatments. *De novo* drug design facilitates the synthesis of novel chemical entities based on known antifungal targets to create highly curated drugs (18). However, *de novo* development can take decades to complete the discovery-to-trial pipeline often due to the challenges in production scale-up, formulation, and the need to chemically modify compounds to reduce cellular toxicity and improve pharmacokinetics and pharmacodynamics (19). To circumvent these issues of *de novo* drug development, investigational and FDA-approved drugs curated into easily accessible libraries (20, 21) can be functionally repurposed for novel uses to reduce the time, cost, and efforts to develop new antifungals (22–25). Here, we report the adaptation and optimization of a rapid tetrazolium salt reduction assay using 2,3-bis(2-methoxy-4-nitro-5-sulfo-phenyl)-2H-tetrazolium-5-carboxanilide (XTT) in a conventional 96-well microtiter plate format to identify antifungal agents effective against spherule growth from four libraries: the Broad Repurposing Hub (26), Prestwick Chemical 1520, Selleck L8200 Anti-parasitic, and MedChemExpress CNS Penetrants libraries.

## RESULTS

### Development and optimization of a screening platform to identify drugs with inhibitory activity against *C. posadasii* spherules

**Fig 1:**
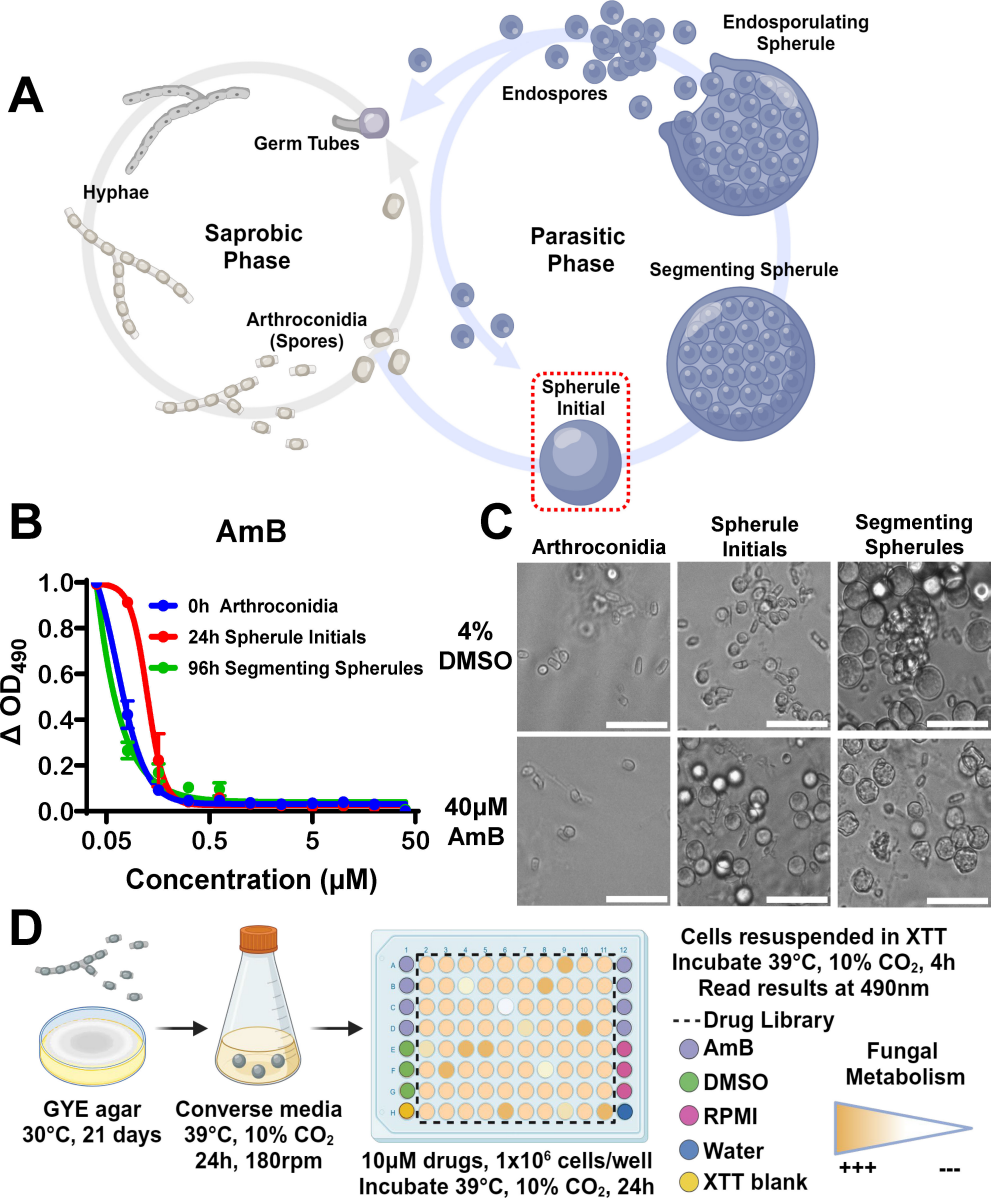
Illustrations of the lifecycle and a screening method for metabolic inhibitors of *Coccidioides*. (**A**) Diagram of the biphasic lifecycle of *Coccidioides*. Arthroconidia (spores) are aerosolized by soil disturbance, and their inhalation initiates a switch from a saprobic to a parasitic phase, where instead of hyphae, spores develop into spherule initials containing a large vacuole (red box) that mature into large segmenting spherules filled with endospores, which continue the parasitic cycle. (**B**) Dose–response curve of 0 h arthroconidia, 24 h spherule initials, and 96 h segmenting spherules exposed to amphotericin B (AmB) for 24 h. (**C**) Brightfield images of each morphology after 24 h of exposure to DMSO or AmB; 400× magnification, scale bar = 100 µm. (**D**) Schematic of the colorimetric assay used to screen spherule initials against the drug libraries.

*Coccidioides* is characterized by a unique spherule morphology, as illustrated in Fig. 1A. To assess if these distinct morphologies exhibited similar drug susceptibilities to AmB, we exposed arthroconidia, spherule initials, and segmenting spherules to a serial dilution of AmB (Fig. 1B). The 50% inhibitory concentration (IC_50_) of arthroconidia (0.06 µM) was most similar to segmenting spherules (0.01 µM), while spherule initials were twofold less susceptible to AmB with an IC_50_ of 0.12 µM. The phenotypic impacts of AmB on each morphology are demonstrated in Fig. 1C. Notably, spherule growth and differentiation are asynchronous, so small populations of earlier morphologies are still present in the media after incubation. Spherule initials were selected as our ideal screening morphology over arthroconidia and mature segmenting spherules due to their clinical relevance and short isotropic growth period, and as the initial stage of the parasitic cycle, these spherule initials constitute a promising target to prevent downstream propagation (27). Using spherule initials as target cells, we developed an anti-*Coccidioides* drug screening platform based on metabolic inhibition (Fig. 1D). Briefly, spherule initials were inoculated into 96-well plates in the presence of 10 µM of screening compound, dimethylsulfoxide (DMSO)-negative controls, AmB-positive controls, or media alone. After a 24 h incubation, the drug supernatant was removed, and cells were resuspended in XTT, a colorimetric indicator of cell viability, for 4 h to reach the optimum OD_490_ for reading absorbance.

### Identification of drugs with novel activity against *Coccidioides*

**Fig 2:**
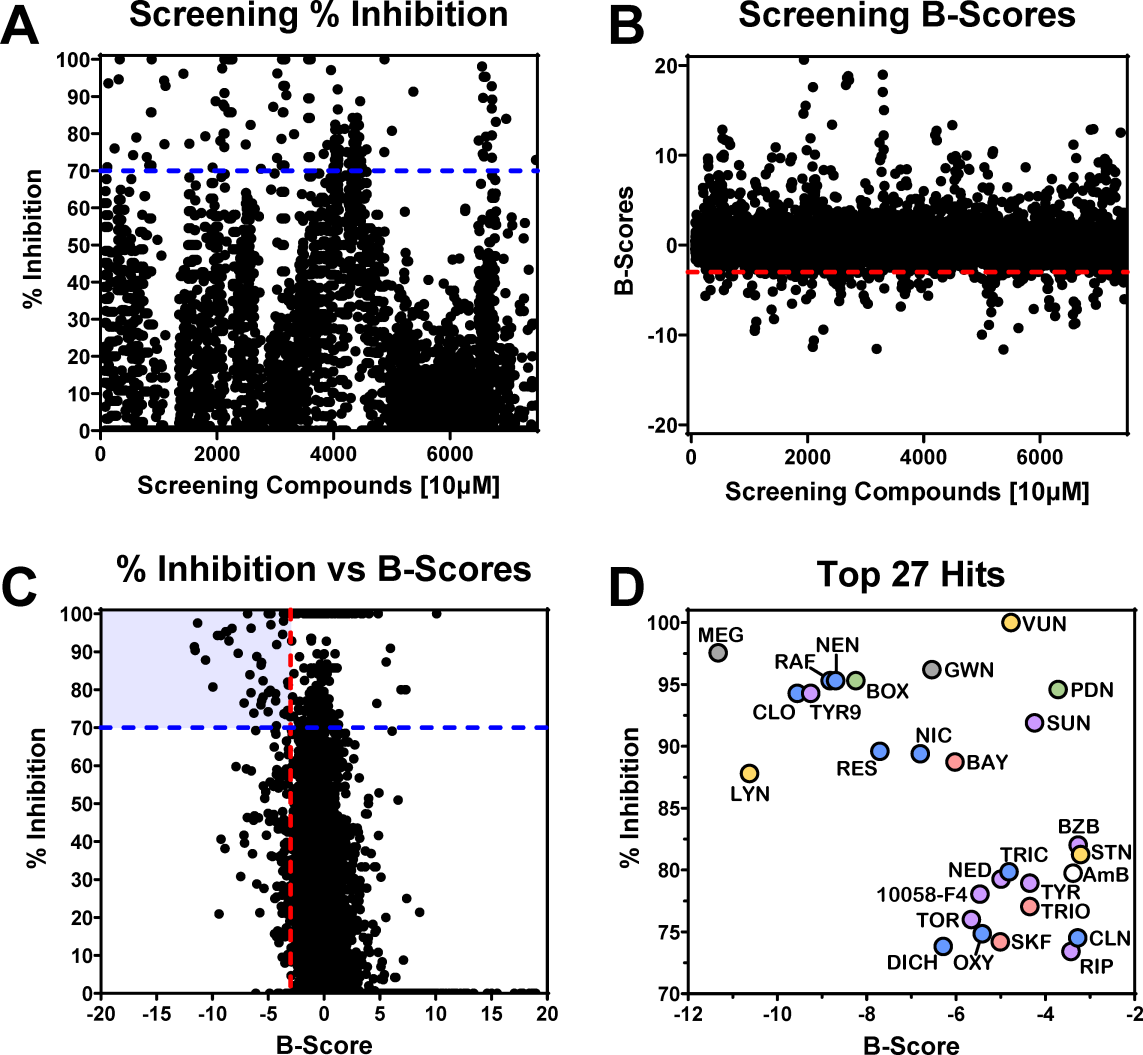
Screening of 7,722 (10 μM) compounds against C735 spherule initials yields 27 novel *anti-Coccidioides* compounds. (**A**) Drug screening results expressed in percent inhibition. A total of 254 drugs were found to inhibit spherule metabolism greater than 70% (blue line). (**B**) Screening B-scores relative to each plate. A total of 172 drugs had B-scores less than −3 (red line). (**C**) Screening B-scores plotted against percent inhibition. Forty-two unique compounds exhibited both a percent inhibition greater than 70% (blue line) and a B-score less than −3 (red line). Thirteen compounds were removed from downstream analysis due to being classified as clinical antifungals and known toxins or because they were not available to purchase. Twenty-seven compounds advanced for further exploration. (**D**) An enhanced view of the upper left shaded gray box containing the top 27 hit compounds. General drug classifications are represented as follows: anti-inflammatory (red; *n* = 4), neurologics (yellow; *n* = 3), analgesics (green; *n* = 3), anti-infectives (blue; *n* = 10), and miscellaneous (gray; *n* = 2). Library screenings were performed in singlet.

A total of 7,722 compounds from our four drug libraries were tested using the XTT assay described above. Percent inhibition was calculated by normalizing the colorimetric readouts of each well to the average DMSO controls, and hits were identified as those with ≥70% growth inhibition. Data were further normalized using B-scores, a statistical analysis accounting for daily and positional variability in drug screenings (28, 29). Overall, we identified 254 hits (3.29% hit rate) showing ≥70% inhibition (Fig. 2A; Fig. S1) and 172 hits (2.22% hit rate) when using the calculated B-scores ≤ −3 (Fig. 2B). A total of 42 compounds (0.54% hit rate) in the initial screening met both B-score and percent inhibition criteria (Fig. 2C). After parsing out any toxins, clinically approved antifungals, and Food and Drug Administration (FDA)-withdrawn compounds, 27 diverse drugs (0.35% hit rate) from a variety of classes and clinical applications remained, and their percent inhibition versus B-scores were plotted in Fig. 2D and listed in Table 1. In addition to our 27 hits, we evaluated three drugs identified by our group in previous repurposing screenings against *Candida albicans* and *Cryptococcus neoformans*, which were present in our library but not captured by our criteria, including pentamidine isethionate (PENT-I) (30), auranofin (AUR) (16), and oxethazaine (OXE) (31).

**TABLE 1:** Top 30 hit compounds ranked by IC50

Rank	Drug name	Acronym	PubChem CID #	General drug classification*	Therapeutic sub-classification	Screening % inhibition	Screening B-score	IC50 (µM)	IC50 (µg/mL)
CTRL	Amphotericin B	AmB	5280965	Anti-infective	Polyene antifungal	79.74	−3.38	0.04	0.04
CTRL	Fluconazole	FLU	3365	Anti-infective	Triazole antifungal	ND^*a*^	ND	8.98	2.75
CTRL	Caspofungin acetate	CAS	6850808	Anti-infective	Echinocandin antifungal	ND	ND	14.61	17.73
CTRL	Dimethyl sulfoxide	DMSO	679	Miscellaneous	Solvent	ND	ND	ND	ND
1	Niclosamide ethanolamine	NEN	14992	Anti-infective	Anti-parasitic	95.30	−8.70	0.55	0.21
2	Niclosamide	NIC	4477	Anti-infective	Anti-parasitic	89.40	−6.80	0.74	0.24
3	Tyrphostin-A9	TYR9	5614	Anti-neoplastic	Tyrosine kinase inhibitor	94.29	−9.25	0.90	0.25
4	Rafoxanide	RAF	31475	Anti-infective	Anti-parasitic	95.30	−8.82	1.25	0.78
5	Dichlorophene	DICH	3037	Anti-infective	Anti-parasitic	73.83	−6.29	1.48	0.40
6	Pentamidine isethionate	PENT-I	8813	Anti-infective	Anti-parasitic, anti-fungal	ND	ND	2.52	1.49
7	10058-F4	10058-F4	1271002	Anti-neoplastic	Apoptosis inducer	78.05	−5.47	3.11	0.78
8	Oxyclozanide	OXY	16779	Anti-infective	Anti-parasitic	74.85	−5.41	3.80	1.53
9	Tyrphostin-AG-879	TYR	135419190	Anti-neoplastic	Tyrosine kinase inhibitor	78.95	−4.35	4.30	1.36
10	SKF-86002	SKF	5228	Anti-inflammatory	Non-steroidal enzyme inhibitors	74.19	−5.01	4.39	1.31
11	Oxethazaine	OXE	4621	Analgesic	Local anesthetic	ND	ND	4.65	2.17
12	Bortezomib	BZB	387447	Anti-neoplastic	Proteasome inhibitor	82.02	−3.27	5.85	2.25
13	SU014813	SUN	10138259	Anti-neoplastic	Tyrosine kinase inhibitor	91.89	−4.24	6.53	2.89
14	BAY-11–7082	BAY	5353431	Anti-inflammatory	Non-steroidal antiinflammatory	88.73	−6.02	7.27	1.51
15	Closantel Sodium	CLN	23683671	Anti-infective	Anti-parasitic	74.52	−3.28	7.37	5.05
16	VU-0422288	VUN	73058507	Neurologic	Glutamatergic inhibition	100.00	−4.77	7.91	2.85
17	Triamcinolone	TRIO	31307	Anti-inflammatory	Corticosteroid	77.05	−4.35	8.06	3.18
18	Triclabendazole	TRIC	50248	Anti-infective	Anti-parasitic	79.86	−4.82	8.25	2.97
19	PD-198306	PDN	9956637	Analgesic	Anti-hyperalgesic	94.59	−3.72	8.26	3.93
20	ST-1859	STN	14187	Neurologic	Anti-amyloidogenic	81.25	−3.21	8.30	2.49
21	LY310762	LYN	11957576	Neurologic	Serotonergic modulator	87.80	−10.62	9.05	3.90
22	Toremifene	TOR	3005573	Anti-neoplastic	Estrogen receptor modulator	76.00	−5.66	9.35	5.59
23	Auranofin	AUR	16667669	Anti-inflammatory	Anti-rheumatic	ND	ND	9.54	6.47
24	Closantel	CLO	42574	Anti-infective	Anti-parasitic	94.29	−9.55	9.72	6.45
25	Bardoxolone	BOX	400010	Analgesic	Enzyme inhibitor	96.13	−8.24	10.46	5.14
26	Meglumine	MEG	8567	Miscellaneous	Hexosamine excipient	97.56	−11.33	11.59	2.26
27	DCC-2618	RIP	71584930	Anti-neoplastic	Tyrosine kinase inhibitor	73.42	−3.43	17.94	9.16
28	Resorantel	RES	65696	Anti-infective	Anti-parasitic	89.60	−7.71	>40	>12.33
29	GW-501516	GWN	6917890	Miscellaneous	Dyslipidemic	79.27	−4.99	>40	>18.14
30	Nedaplatin	NED	9803963	Anti-neoplastic	Apoptosis inducer	96.20	−6.54	>40	>12.13

^*a*^ ND, not determined.

### Concentration-dependent assays for hit confirmation and potency determination

**Fig 3:**
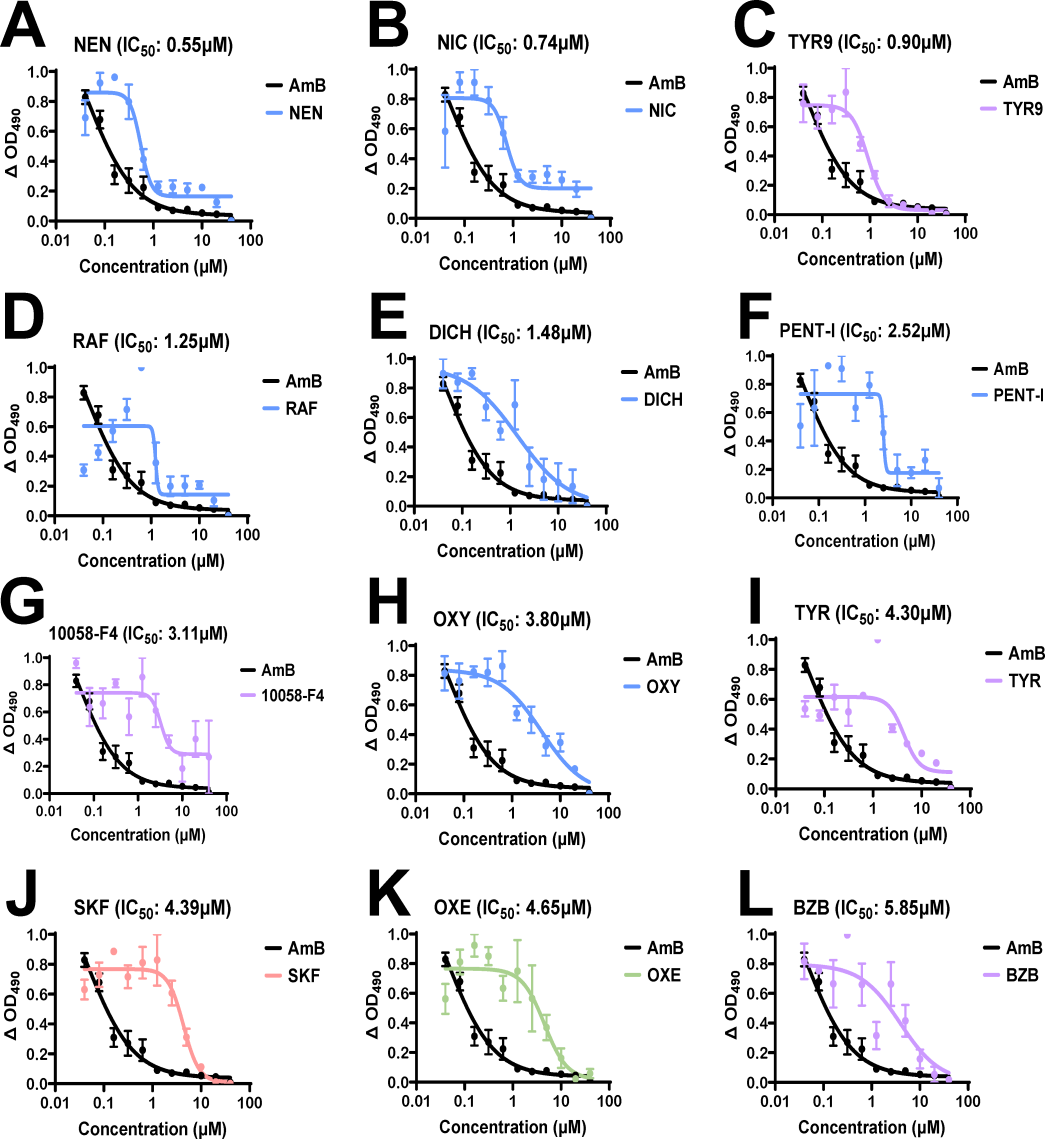
Dose–response curves of top 12 compounds with IC_50_ values < 6 µM. (**A–L**) Dose–response curves of drugs with IC_50_ values < 6 µM against 24 h spherule initials compared to the amphotericin B control (black curve). The color of each curve corresponds to the following drug classifications: red—anti-inflammatory; green—analgesic; blue—anti-infective; and purple—antineoplastic. IC_50_ values were determined using a four-parameter variable-slope nonlinear regression and fractionally normalized. Data are presented as means ± standard error of the mean from triplicate experiments.

We conducted concentration-dependent assays to confirm and establish the potency of the 30 down-selected compounds. We determined IC_50_ by testing the spherule initials against a two-fold dilution series over 40 to 0.04 µM. The IC_50_ values for 27 drugs ranged between 0.55 and 17.94 µM (Fig. 3; Fig. S2; Table 1). We classified these compounds into six general application groups according to the PubChem database: anti-inflammatories (*n* = 4), neurologics (*n* = 3), analgesics (*n* = 3), anti-infectives (*n* = 10), anti-neoplastics (*n* = 8), and miscellaneous (*n* = 2). The 30 potential candidates, their known therapeutic indications, and their screening scores are shown in Table 1. Overall, 12 promising compounds demonstrating low-micromolar IC_50_ values all below 6 µM were down-selected for further characterization.

### Five compounds demonstrate synergy with amphotericin B

**Fig 4:**
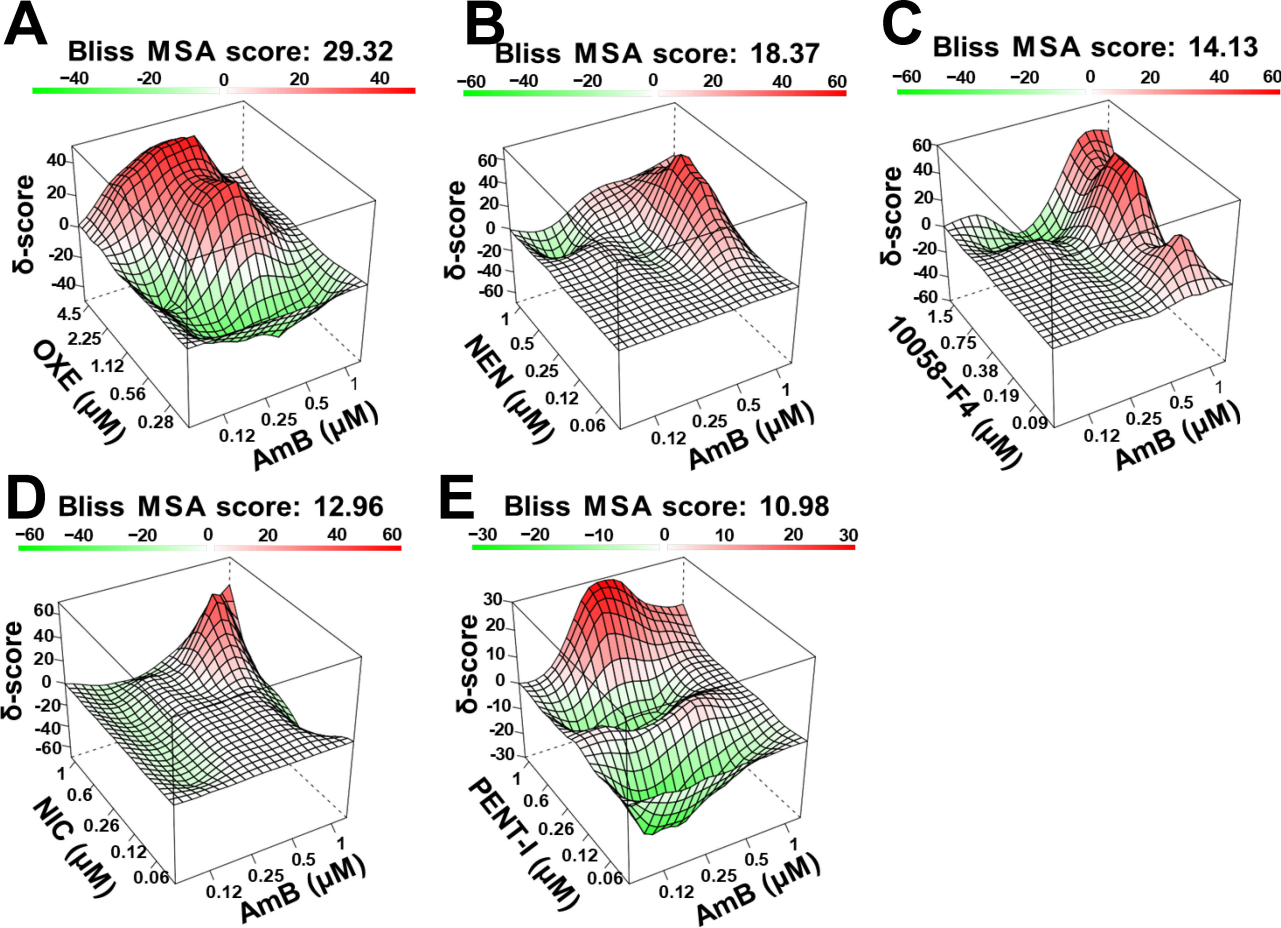
Five drugs inhibit *C. posadasii* growth synergistically when combined with amphotericin B. (**A–E**) Topographical synergy maps of the top five drugs combined with AmB highlight synergistic (red) and antagonistic (green) dose regions. Bliss synergy scores are represented on the *Y*-axis as the delta score (δ-score), while the *X*- and *Z*-axes depict the combined concentration of each drug. The most synergetic area (MSA) score indicating the highest consensus synergy score within a 3 × 3 dose window (red peak) is listed above each graph.

We further investigated whether the 12 down-selected compounds would demonstrate synergistic effects (enhanced cooperative effects) when combined with AmB, FLU, and CAS in checkerboard synergy assays. The Bliss independence model was used for the statistical analysis. Bliss most synergistic area (MSA) scores were calculated for each combined pair of drugs using Synergy Finder 3.0 (32). MSA scores are defined as synergistic (≥10), additive or indifferent (−10 to 10), or antagonistic (≤−10) (Table 2). The concentration ranges tested were a 2× dilution step above each drug’s IC_50_. Five compounds displayed synergistic MSA scores with AmB ranging between 10.98 and 29.32, including OXE (analgesic), NEN (anti-parasitic), 10058-F4 (apoptosis inducer), NIC (anti-parasitic), and PENT-I (anti-parasitic) (Fig. 4A through E). The scores are summarized in Table 2 where, notably, none of the compounds showed synergy with FLU or CAS. Other notable antagonistic effects observed included SKF with AmB, TYR9 with FLU, and NIC with CAS. The chemical structures of AmB, FLU, CAS, and the five drugs synergistic with AmB are shown in Fig. S3A through H. The five drugs demonstrate low structural similarity scores (<0.8) when compared with AmB, FLU, and CAS, indicating structural distinction from the current coccidioidomycosis treatments (Fig. S3I).

**TABLE 2:** Bliss synergy table of top 12 compound interactions

Acronym	Tested range (µM)	Bliss MSA scores^*a*^		
		AmB (2–0.13 µM)	FLU (16–0.5 µM)	CAS (36–1.13 µM)
OXE	9–0.28	29.32	−6.86	6.13
NEN	2–0.06	18.37	0.35	0.00
10058-F4	3–0.09	14.13	−5.74	4.58
NIC	2–0.06	12.96	−6.58	−10.39
PENT-I	2–0.06	10.98	1.59	0.00
TYR	9–0.28	7.94	−9.80	−1.81
DICH	4–0.13	4.24	6.69	3.75
RAF	2–0.06	2.25	−0.74	7.10
TYR9	2–0.06	−1.85	−11.95	3.21
OXY	7–0.22	−4.68	−1.14	−6.95
BZB	10–0.31	−5.11	−0.24	−2.85
SKF	9–0.28	−10.09	6.84	6.84

^*a*^ MSA: most synergetic area in a 3 × 3 dose window in the matrix.

### Impacts of the five leading compounds on cell morphology

**Fig 5:**
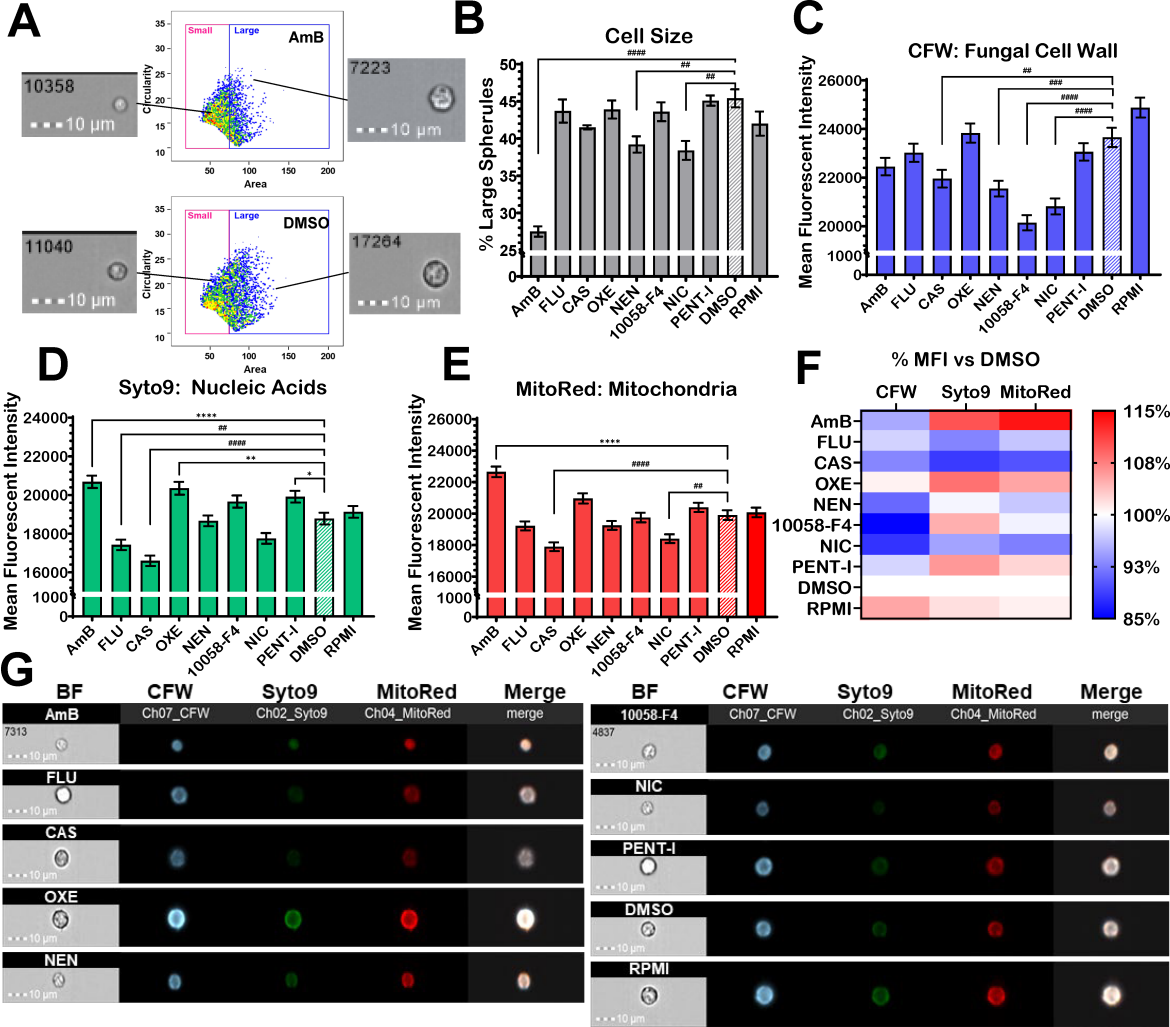
Cell profiling of 24 h, 20 µM drug-treated spherule initials by image flow cytometry. (**A**) Scatterplot of circularity against area demonstrates how cells were defined as small (<75 µm^2^) or large (≥75 µm^2^). Representative images from the positive (AmB) and negative (DMSO) controls show spherules from each population. (**B**) Percent large spherules. (**C–E**) Mean fluorescent intensities (MFI) of spherules labeled with calcofluor white (CFW), Syto9, and Mitotracker Red CMXros (MitoRed) to observe alterations to the fungal cell wall, nucleic acid content, and mitochondrial oxidation, respectively. * represents positive increases in MFI, while ^#^ signifies reduction in MFI compared to the DMSO control. (**F**) Heatmap demonstrates a summary of the mean fluorescent intensities of panels C through E as percent of DMSO. Dissimilarities suggest the drugs act by mechanisms unlike conventional antifungals, including AmB, FLU, and CAS. (**G**) Representative images of spherules impacted by each compound. *P < 0.05; ****P < 0.0001 (one-way ANOVA vs DMSO; standard error of the mean error bars, triplicate experiments).

We captured drug-induced alterations to spherule phenotypes using image flow cytometry. Spherule initials were incubated with 20 µM of drug, AmB, FLU, or CAS for 24 h to assess their effects compared to spherules treated with DMSO or media controls. The treated cells were stained with calcofluor white (CFW; fungal cell wall chitin and glucans), Syto9 (nucleic acids), and MitoTracker Red CMXRos (MitoRed; oxidized mitochondria) prior to fixation. Over 2,000 cells with focused images per sample were analyzed using the gating strategy in Fig. S4. Large spherules were defined as circular cells with an area greater than 75 µm^2^ as shown on the scatter plots of representative AmB- and DMSO-treated samples (Fig. 5A). The percentages of large spherules for NEN- and NIC-treated samples were significantly reduced compared to DMSO-treated cells (Fig. 5B; ^#^*P* < 0.01, one-way analysis of variance [ANOVA]). NEN is an ethanolamine salt of NIC, thus both inhibiting spherule growth. NIC-treated cells had significantly reduced the mean fluorescent intensity (MFI) of CFW and MitoRed, similar to CAS-treated cells. However, NIC-treated cells did not reduce the MFI values of Syto9, which were different from CAS-treated cells. NEN- and 10058-F4-treated cells also reduced the MFI of CFW (Fig. 5C and D; ^#^*P* < 0.01, one-way ANOVA). Interestingly, OXE- and PENT-I treated cells showed significantly elevated levels of MFI-Syto9 (Fig. 5C and D; *P < 0.01, one-way ANOVA). Altogether, these data showed that the five lead compounds impact spherule components with unique labeling patterns as shown in Fig. 5F and G, and these patterns were also distinct from AmB, FLU, and CAS.

### Common hits were identified for both virulent *C.p.* C735 isolate and an attenuated vaccine strain (ΔT)

**Fig 6:**
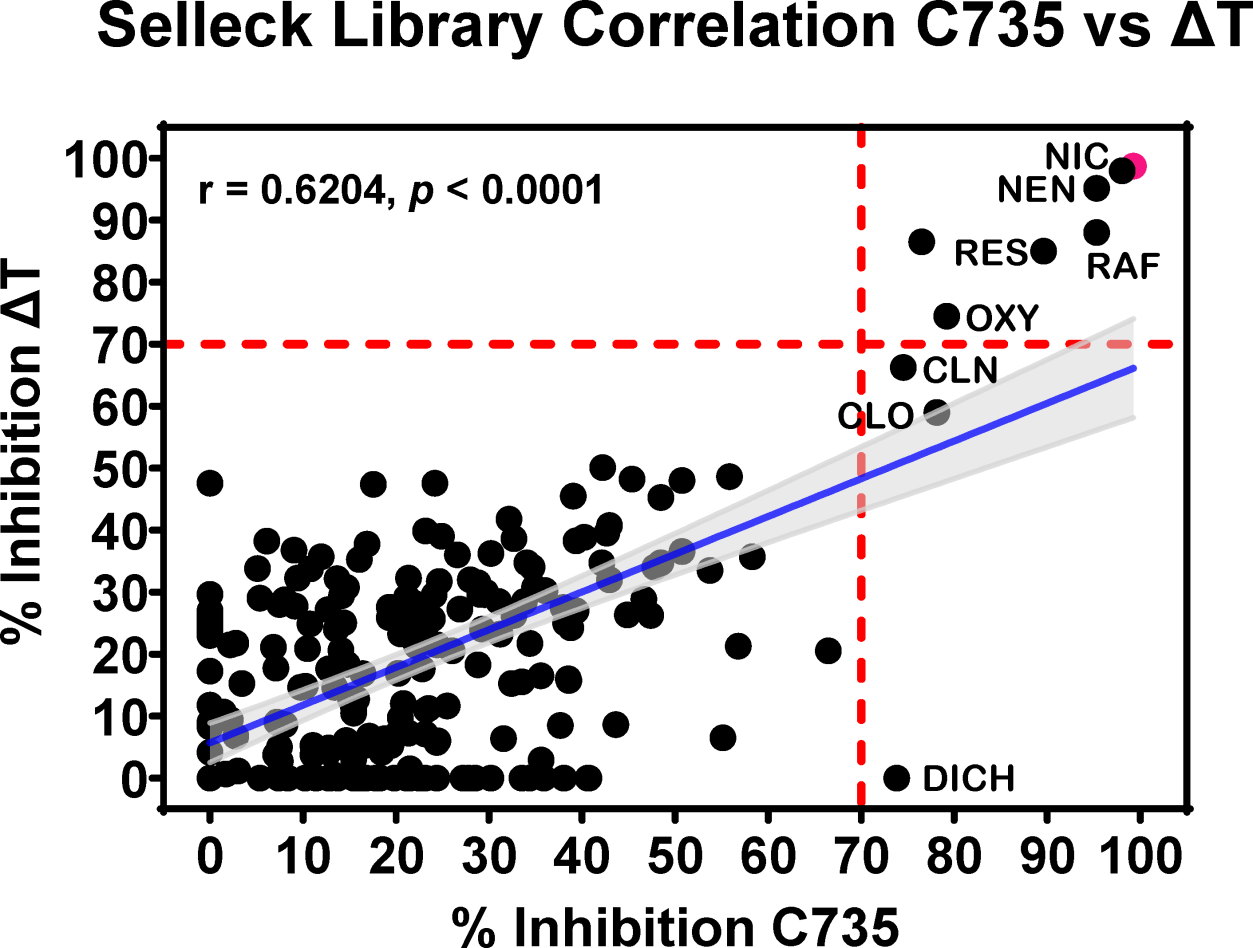
Attenuated ∆T strain can act as a drug-screening surrogate. As a proof-of-concept, the avirulent *C. posadasii* C735 *∆cts2/ard1/cts3* triple mutant (∆T) was screened against the Selleck library, and its results were compared to those of the parental *C. posadasii* C735 strain. The blue line is a simple linear regression of the Pearson correlation coefficient (*r*), and the gray lines represent 95% confidence intervals. The AmB-positive control is highlighted in pink. The dashed lines (red) at 70% inhibition indicate our cutoff for defining drugs as a hit compound.

The ∆T mutant was genetically engineered by deleting the expression of two chitinase genes (*cts2, cts3*) and truncating the 3′ end of arabinol-2-dehydrogenase 1 (*ard1*), a gene located next to *CTS3* of the *C.p*. C735 isolate (33). The ∆T strain displays normal hyphal growth and forms morphologically typical arthroconidia, which can convert to spherule initials; however, ∆T abolishes endosporulation and cannot complete the parasitic cycle (33). Mice inoculated with 1 × 10^6^ viable ∆T spores by the intratracheal route thrice over a 2 week interval are healthy with no sign of sickness, while the LD_100_ of the parental *C.p*. C735 is <100 spores. ∆T can be cultured and manipulated in a BSL-2 laboratory, which is a more conventional research environment for drug discovery. We evaluated whether the ∆T could be used as a surrogate strain for drug screening using the Selleck L8200 Library. The XTT assays identified eight compounds that inhibited the growth of both the ΔT mutant and the *C.p.* C735 parenteral strain (Fig. 6; Table 1). The overall correlation of the growth inhibition activity against the C735 virulent and the ∆T was moderate with a Pearson’s correlation coefficient value (*r*) of 0.62 (*P* < 0.0001). These results suggest that the ΔT attenuated mutant may serve as a surrogate strain for screening anti-*Coccidioides* drugs in a BSL-2 laboratory, which can then be confirmed in a virulent strain.

## DISCUSSION

Our XTT colorimetric assay for screening drug libraries using *Coccidioides* spherule initials enhances the current antifungal screening framework for human pathogenic fungi and complements the existing drug screening by Mead et al. (34). Mead et al. performed duplicate screenings of the 1,280-compound LOPAC library at 1 and 5 µM, identifying compounds with >50% growth inhibition against ∆T arthroconidia over a 120 h period at 30°C by measuring OD_600_ every 24 h. Mead et al. identified some overlapping hits (Table 1), and their average IC_50_ were NIC (1.02 µM), TYR9 (1.36 µM), AUR (3.53 µM), TYR (10.82 µM), and PENT-I (34.5 µM). Identifying similar hits in these independent assays validates our screening methodology, with the added advantage of a faster screening platform targeting the clinically relevant spherule initial morphology, which is crucial for disease control.

XTT was an ideal screening indicator due to its cell wall permeating ability, short incubation time, and the readout simplicity. Additionally, for spherule initials, a metabolic readout was preferred over OD_600_ for spherules given that, unlike *Saccharomyces cerevisiae*, whose generation time is 2 h, *Coccidioides* spherules grow radially until releasing endospores around day 5, making it challenging to quantify population growth. Our screening identified several compounds with significant antifungal activity, with 254 drugs showing more than a 70% reduction in spherule metabolism. We implemented a B-score cutoff below −3 to stringently parse these hits for the most potent antifungal agents. Only 27 compounds met both our percent inhibition and B-score criteria, exhibiting varying degrees of potency, with IC_50_ values ranging from 0.55 to 17.94 µM (0.21 to 9.18 µg/mL, respectively). Twelve hits demonstrated IC_50_ under 6 µM. These findings underscore the feasibility of using XTT assays in drug discovery for *Coccidioides.*

We conducted a combination study using three common systemic antifungals: AmB, FLU, and CAS. AmB demonstrated strong synergy with five of the 12 drugs: OXE, NEN, 10058-F4, NIC, and PENT-I. To our knowledge, we are the first to demonstrate these five drugs’ abilities to potentiate AmB activity against *Coccidioides*. As these five drugs have been noted in primary screenings of other fungal pathogens, this highlights their additional potential to be repurposed as broad-spectrum antifungals.

To further investigate the antifungal effects and potential mechanisms of action of these five synergistic drugs, we employed image flow cytometry (31). By combining quantitative analysis with microscopy, this technique can capture drug-induced phenotypic changes, which may elucidate these drugs’ mechanisms of action in terms of how similar or distinct they act compared to controls.

OXE or oxetacaine is a local anesthetic and antacid in the treatment of peptic ulcers with antifungal activity against *Cryptococcus neoformans* based on morphologic impacts seen in image flow cytometry by McMahon et al. (31). In our study, OXE-treated cells showed increased Syto9 fluorescent intensity similar to AmB, suggesting enhanced nucleic acid content or accessibility, possibly by interfering with nucleic acid processes. 10058-F4 is an antineoplastic agent thought to induce apoptosis in mammalian cells by inhibiting the c-Myc signal pathway; it was discovered to have fungicidal activity against *C. neoformans* in Rabjohns et al.’s screening of the LOPAC under nutrient-limiting conditions (35). Our results showed that 10058-F4-treated spherules had significantly decreased CFW fluorescence compared to DMSO, which may be consistent with its role in inducing cellular stress and apoptosis.

PENT-I or nubapent is an anti-parasitic for leishmaniasis and trypanosomiasis that can be nebulized to treat *Pneumocystis* pneumonia (36) and has shown some efficacy against *Candida auris* (30). In our study, PENT-I-treated cells exhibited moderately increased Syto9 fluorescence, which may indicate alterations in nucleic acid content or cell permeability.

Our most efficacious drugs overall, NIC, and its more soluble ethanolamine salt, NEN, are aromatic anilides historically used as anticestodals for helminth infections. NIC and NEN have strong pan-antifungal drug potential, as NIC is reportedly effective against several pathogenic fungi, including *Histoplasma capsulatum* (37), *Paracoccidioides brasiliensis* (37), *Sporothrix brasiliensis* (37), *C. albicans* (38), and *C. neoformans* (31), while both NIC and NEN can prevent eumycetoma development by *Madurella mycetomatis* (39). Both NIC- and NEN-treated cells in our study significantly decreased cell size, similar to AmB, while they also reduced CFW without affecting Syto9. Interestingly, only NIC demonstrated additional reduction in MitoRed staining. These data suggest that these drugs disrupt the cell wall and mitochondrial activity, consistent with their broad antifungal activity.

NIC is well-tolerated in humans, as the adult treatment dose for gastrointestinal helminths is a 2 g oral dose administered once daily for 7 days (40). Due to the hydrophobic nature of these drugs, this regimen is not suitable for pulmonary or systemic fungal infections. There have been efforts by Sutar et al. (38) to develop oral and mucosal formulations of niclosamide-loaded nanoparticles for treating murine models of oropharyngeal and vulvovaginal candidiasis; however, efficacy against coccidioidomycosis would require the development of systemic formulations.

Our results reveal that the cytological profiles (i.e., size, fluorochrome binding to cell wall, DNA/RNA, and mitochondria) of spherules treated with the top five drugs are distinct from those treated with AmB, FLU, or CAS, suggesting these drugs act through unique mechanisms. Drugs acting through different cellular functions that inhibit parallel pathways are advantageous since they are less vulnerable to antifungal resistance, a major global concern (41). Although the precise MOA of these drugs was not captured by this basic screening (42), more sensitive and specific techniques like RNA-seq and targeted mutagenesis will be essential to precisely defining these drugs’ mechanisms of action in *Coccidioides*.

In our screening of the Selleck Library against C735 and ΔT, we identified a novel class of anti-*Coccidioides* compounds, called the halogenated salicylanilides, a group of anti-parasitic protonophores, which include NIC and its ethanolamine salt (NEN), rafoxanide (RAF), oxyclozanide (OXY), closantel sodium (CLN), closantel (CLO), and resorantel (RES), which were all captured in our screening (Table 1). Since NIC and NEN have been demonstrably potent, this further highlights that additional investigation of the halogenated salicylanilides is warranted. Most excitingly, this hit correlation between the parental and mutant suggests that ΔT can act as a surrogate for drug screening in a BSL-2 environment, and then be confirmed in a virulent strain, which would greatly increase the accessibility of anti-*Coccidioides* drug screening.

The two species, *C. posadasii* and *C. immitis*, are epidemiologically and genetically distinct, having diverged approximately 5.1 Mya (19). However, they are morphologically and clinically identical with similar virulence (43). Both species grow in semi-desert soils as hyphal mats within endemic areas in conditions of high salinity and high temperature (44). Although *C. posadasii* hyphae grow faster than *C. immitis* at 37°C, the differences in parasitic growth remain poorly investigated (45). These growth, genetic, and protein variations could lead to variations in drug efficacy between the species or isolates, which remain to be explored, especially between the two species and drug-resistant strains.

In summary, we have developed and optimized a BSL-3 screening methodology to identify compounds active against *Coccidioides* spherule initials and evaluated their *in vitro* potency, synergistic activity with systemic antifungals, and effects on spherule phenotypes. Notably, five compounds (OXE, NEN, 10058-F4, NIC, PENT-I) demonstrated potent IC_50_ values and synergistic effects with AmB. We also showed that an attenuated BSL-2 mutant can serve as a drug screening surrogate, expanding the accessibility of these tools and assays to BSL-2 laboratories, which we hope will further facilitate the discovery of anti-*Coccidioides* therapeutics within the field for the betterment of the valley fever community.

## MATERIALS AND METHODS

### Fungal cultures

The clinical isolate *Coccidioides posadasii* C735 and its genetically engineered attenuated mutant *C. posadasii* C735 ∆*cts2*/*ard1*/*cts3* (∆T) were used in this study (33). C735 was handled in a BSL-3 laboratory, while the ∆T strain was managed in a BSL-2 laboratory. *Coccidioides* arthroconidia were harvested after growing for 3–4 weeks on glucose yeast extract agar plates (1% glucose, 0.5% yeast extract, 1.5% agar) that were incubated at 30°C. Converse medium was inoculated at a density of 2–5 × 10^8^ arthroconidia per 100 mL as previously described (46). To obtain spherule initials, cultures were incubated at 39°C, 10% CO_2_, and 180 rpm for 24 h, while segmenting spherules utilized the same conditions over 96 h. Spherules were collected by centrifugation at 2,000 rpm for 10 min at room temperature and adjusted to the desired final density using 2.0 g/L sodium bicarbonate-buffered Roswell Park Memorial Institute medium (RPMI) 1640 without phenol red (Gibco, #11835030).

### Chemical libraries and compounds

Several repurposing drug libraries containing diverse mixes of investigational and FDA-approved/registered compounds were utilized for this study, including the Broad Institute Repurposing Library (5,200 compounds), the Prestwick Chemicals Library (1,520 compounds), the Selleck L8200 Antiparasitic Library (219 compounds), the MedChemExpress HY-L028 CNS Penetrants Library (782 compounds), and the compound niclosamide ethanolamine (Adipogen, AG-CR1-3644-M100), totalling to 7,722 compounds. Compounds from the Broad Library were pre-spotted in 4 µL drops in 96-well plates. The Prestwick, Selleck, and MCE Libraries were supplied as 10 mM stock solutions in DMSO or deionized water and diluted to 1 mM and stored at −80°C as daughter library sets for subsequent screening experiments. Before screening, compounds were diluted to 20 µM (2× concentration) in RPMI 1640 (Gibco, #11835030) and stored at −20°C. Amphotericin B (10 µM; Sigma, #A2942-100), 2% (vol/vol) DMSO, and RPMI served as positive, vehicle, and negative controls, respectively. For subsequent experiments, milligram quantities of individual compounds were purchased and prepared as 1 mM concentrated stock concentrations in DMSO or deionized water.

### Compound screening

Spherule initials (24 h) were screened against 10 µM of compound in a 200 µL volume, with a final cell density of 1 × 10^6^ spherules per well, except the Broad Library, which used 2.45 × 10^5^ spherules/well. Cultures were incubated at 39°C and 10% CO_2_ for 24 h. Following incubation, drug supernatants were removed by pipette or centrifugal filtration (Millipore, MSRLN0410) at 2,000 rpm at 22°C for 10 min. Spherule pellets were resuspended in 100 µL of XTT solution (0.5 mg/mL in DPBS; Cat #J61726; Thermo Fisher Scientific) containing 0.07 µM menadione (10 mM stock in acetone; Sigma, M5625). Spherules were incubated in XTT for 4 h at 39°C and 10% CO_2_ spun down at 2,000 rpm for 10 min. After incubation, the supernatant was transferred to a 96-well flat-bottom plate (Fisherbrand, #12565501) to read the 490 nm absorbance (OD_490_) using a BioTek ELx808 absorbance plate reader with Gen5 microplate reader and Imager software v3.10.06 (Agilent BioTek). Screening outliers were filtered using the Knime v4.7.5 (47) outlier removal node, which excluded wells with a 490 nm readout ± 3 standard deviations from the mean plate controls. Of the 9,291 wells screened, 134 wells were excluded as outliers. Percent inhibitions of the remaining drugs were calculated as follows:


$$\%\text{ inhibition}=1-\left[\frac{\lambda_{\text{drug}}-\lambda_{\text{XTT}}}{\lambda_{\text{DMSO}}-\lambda_{\text{XTT}}}\right]\times 100\%,$$

where λ is the OD_490_ value. Percentages were min–max normalized so that any values <0 and >100% were assigned to 0 and 100%, respectively. *B*-scores, a positional-based *Z*-score with a median polish, were calculated using the normalize plate (*B*-score) node as follows (28, 29):


$$Y_{ijp}=\mu_{p}+\rho_{i}+\gamma_{j}+\varepsilon_{ijp},$$

where *Y_ijp_* is the value in the *i*th row and *j*th column of the *p*th plate; µ*_p_* is the plate’s center; ρ*_i_* is the *i*th row effect; γ*_j_* is the *j*th column effect; and ε*_ijp_* is the random noise of the assay on the plate. An iterative median polish is then applied to the row effect, column effect, and overall median. Compounds with ≥70% metabolic inhibition and a *B*-score ≤ −3 were defined as hits. Hits were further qualified for exclusion based on toxicity, carcinogenic activity, FDA withdrawal, or unavailability for further investigation.

### IC_50_ determinations

IC_50_ dose–response assays were performed in triplicate using a 2× serial dilution over a concentration range of 40–0.04 µM in a final volume of 200 µL with a final cell density of 1 × 10^6^ spherule per well following the methodology described in the screening above. IC_50_ values were determined using OD_490_ readouts by a variable-slope four-parameter nonlinear regression model in GraphPad Prism v10.1.0 (GraphPad Software, https://www.graphpad.com/) and calculated using the formula below:


$$Y=\text{bottom}+\frac{\text{top}-\text{bottom}}{1+{\left(\frac{{\text{IC}}_{50}}{X}\right)}^{\text{Hill slope}}},$$

where the half-maximal inhibitory concentration or *IC*_50_ is the calculated variable; *X* is the molar dose concentration; *Y* is the OD_490_ response of a compound; Top and Bottom are the lower and upper bounds of the OD_490_ drug response *Y*, respectively; and Hill slope is the unitless slope coefficient calculated for each drug individually. The lower bound parameter was defined as greater than 0 for these IC_50_ calculations. To maintain the shape of the data but elongate the *Y*-axis for ease of data visualization, the IC_50_ dose–response curve figures plot the triplicate average of fractionally normalized OD_490_ XTT readouts, which bound the OD_490_ between 0 and 1 based on the MIN and MAX values of that individual drug’s data set as below:


$$Y_{\text{fractional}}=\frac{\lambda_{\text{drug}}-\min(\lambda_{\text{drug}})}{\max(\lambda_{\text{drug}})-\min(\lambda_{\text{drug}})}$$

### Drug synergy assays

The checkerboard synergy assays were conducted in triplicate on two 7 × 6 matrices per 96-well plate to measure the interactions of two drug pairs simultaneously. Compounds in synergy assays were dispensed using a liquid handling robot (Assist Plus; Integra, Inc.) using a two-fold dilution protocol. In each matrix, a candidate compound of interest and a single clinical drug (either amphotericin B, fluconazole, or caspofungin) were tested using the optimized XTT assay. The concentration range varied according to the IC_50_ value determined in the dose–response curves, but each well had a final volume of 200 µL with a final cell density of 2 × 10^5^ spherule initials per well.

Bliss synergy scores and the most synergistic areas were determined using Synergy Finder 3.0 (32). Bliss score interpretation is defined as synergistic (>10), additivity or indifferent (−10 to 10), and antagonistic (<−10), respectively.

### Image flow cytometry

Spherule initials were treated with 20 µM of drug, and then were subjected to morphology analysis using an image flow cytometer. Cells were labeled with calcofluor white M2R (CFW) (Sigma, #18909; Ex/Em: 360/430 nm), Syto9 (Invitrogen, S34854; Ex/Em: RNA: 486/501 nm DNA: 485/498 nm), and Mitotracker Red CMXRos (Invitrogen, M7513; Ex/Em: 579/599 nm) with a cocktail of these three dyes at 0.00001%, 0.0005 µM, and 1 µM, respectively, in 50 µL RPMI buffer. Cells were incubated in the dark at 37°C for 20 min, washed with phosphate-buffered saline (PBS) twice, and resuspended in 2% paraformaldehyde (PFA) for 1 h. Cells were washed with Dulbecco's phosphate-buffered saline to remove PFA, and images were analyzed on a five-laser 12-channel Amnis ImageStreamX MKII image flow cytometer using the autosampler. INSPIRE (Amnis, EMD Millipore) software data acquisition parameters were set at 40× magnification over 3 min (7,000 cells/sample) with 405, 488, 561, and 785 nm excitation lasers. Color compensation matrices were determined using single fluorochrome controls. Batch analysis of all acquired compensated images using IDEAS v6.2 software (Amnis, EMD Millipore). Feature values, including mean fluorescent intensity, cell area, aspect ratio (minor/major axes), circularity, percent total, and compactness of each cell within the gated sample population, were exported to Excel and GraphPad Prism for subsequent statistical analysis by one-way ANOVA, where a *P*-value ≤ 0.05 was considered significant. The gating strategy is provided in the supplemental figures.

## SUPPLEMENTAL MATERIAL

Fig. S1 mbio.00205-25-s0001.pdf Raw OD_490_ XTT readouts from library screenings.

Fig. S2 mbio.00205-25-s0002.pdf Dose–response curves of the remaining top 30 compounds.

Fig. S3 mbio.00205-25-s0003.pdf Chemical structures of the top five compounds.

Fig. S4 mbio.00205-25-s0004.pdf Image flow cytometry gating strategy for profiling *Coccidioides posadasii*.

ASM does not own the copyrights to Supplemental Material that may be linked to, or accessed through, an article. The authors have granted ASM a non-exclusive, world-wide license to publish the Supplemental Material files. Please contact the corresponding author directly for reuse.
