# Supplementary material for: Discovery of novel antifungal drugs via screening repurposing libraries against Coccidioides posadasii spherule initials
Source: mBio. 2025 Mar 26;16(5):e00205-25. doi: 10.1128/mbio.00205-25 (PMC12077158; doi:10.1128/mbio.00205-25)
Supplement: Fig. S1 — Raw OD490 XTT readouts from library screenings. [file mbio.00205-25-s0001.pdf]

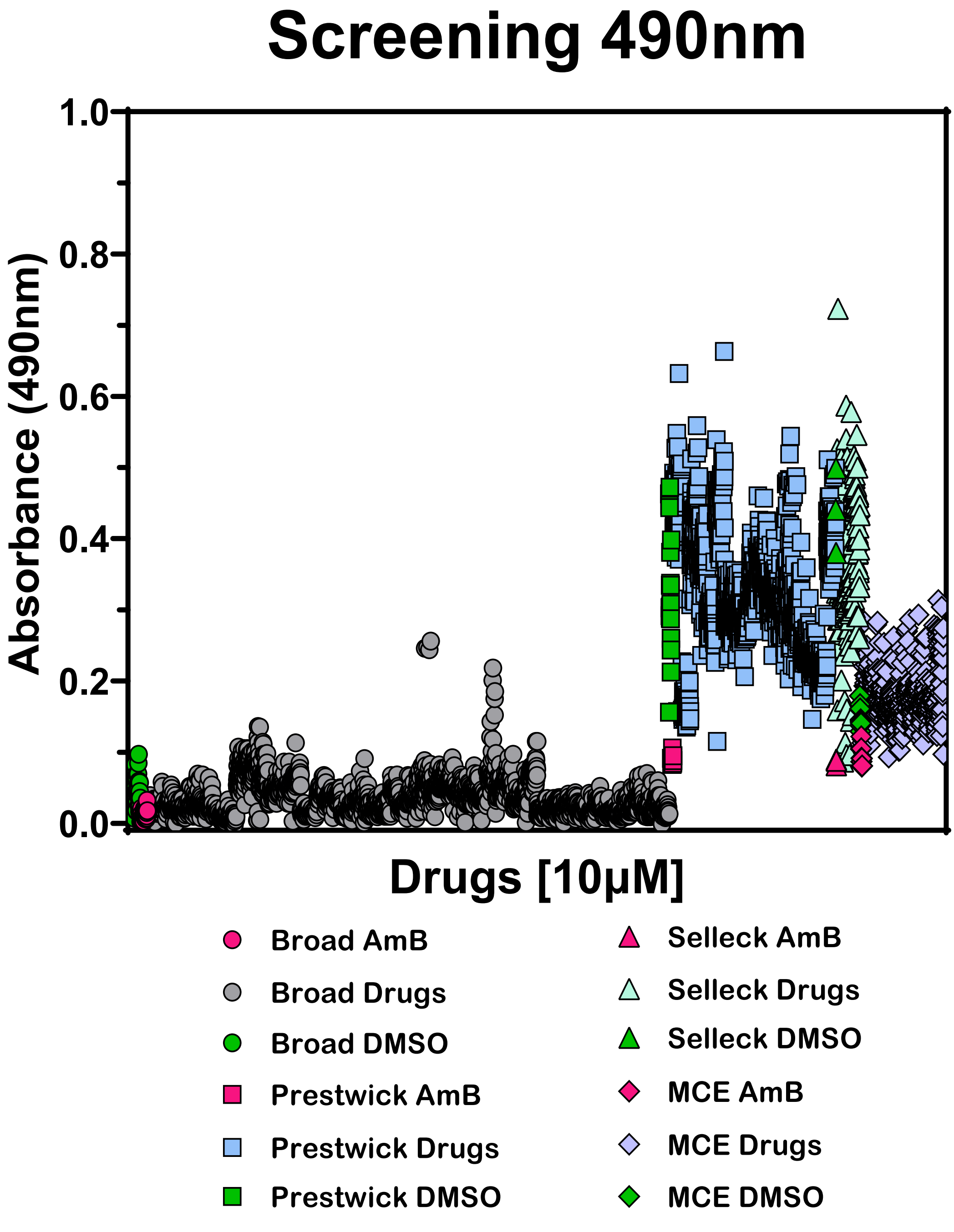

Supplementary Figure 1. Raw OD_{490} XTT readouts from the library screenings. Drug screening XTT results expressed as OD_{490} values. Spherule initials were screened against 10 µM of compound in a 200 µL volume, with a final cell density of $1x10^6$ spherules per well except the Broad Library which used $2.45x10^5$ spherules/well. The higher cell number contributes to a higher OD_{490} value. These OD_{490} values were used to calculate the percent inhibition and B-scores in Figure 2.
