# Supplementary material for: Discovery of novel antifungal drugs via screening repurposing libraries against Coccidioides posadasii spherule initials
Source: mBio. 2025 Mar 26;16(5):e00205-25. doi: 10.1128/mbio.00205-25 (PMC12077158; doi:10.1128/mbio.00205-25)
Supplement: Fig. S2 — Dose–response curves of the remaining top 30 compounds. [file mbio.00205-25-s0002.pdf]

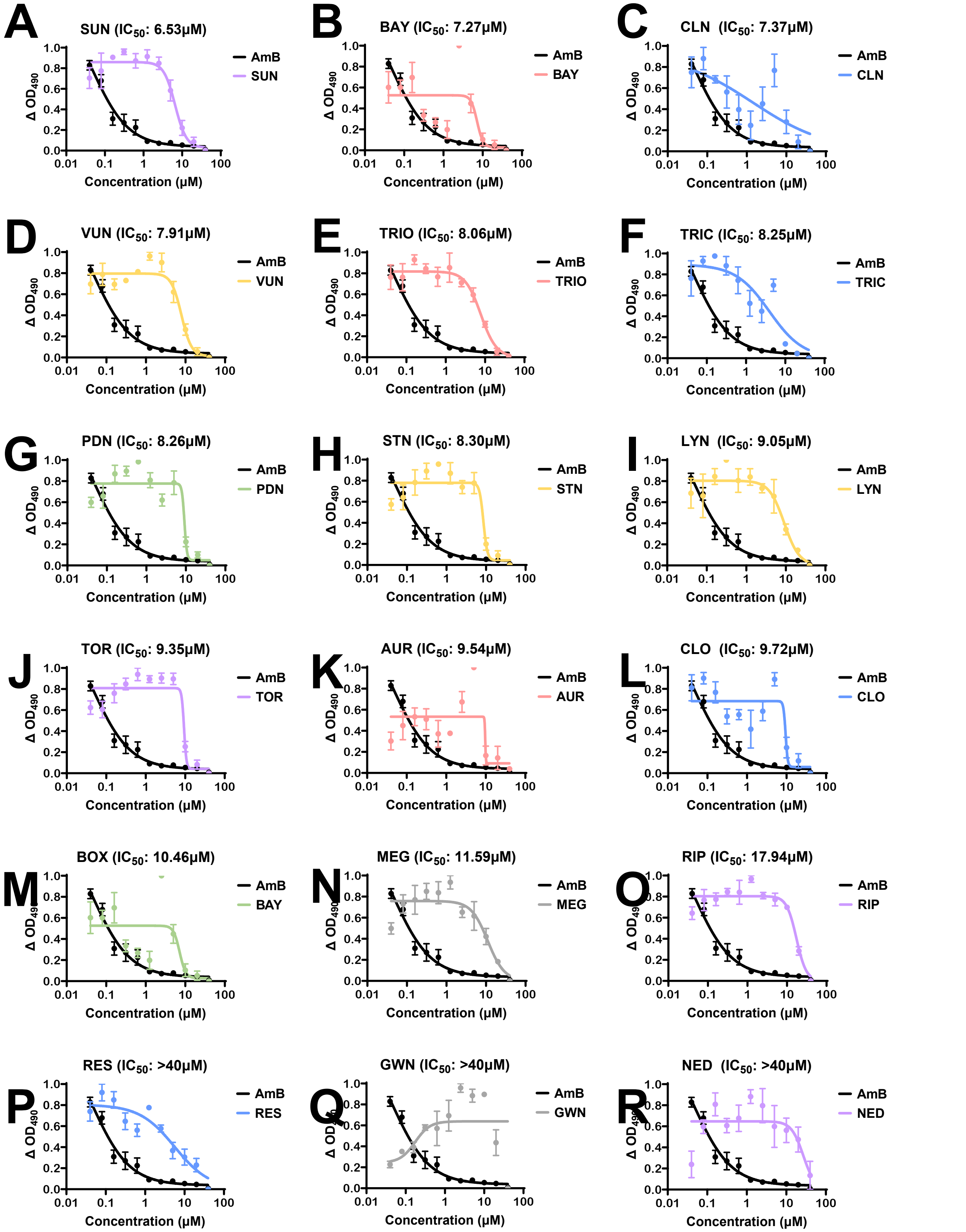

Supplementary Figure 2. Dose response curves of remaining Top 30 compounds. (A-R) Dose-response curves of drugs with IC_{50} values >6 μM against 24-hour spherule initials, compared to the Amphotericin B (AmB) control (black curve). The color of each curve corresponds to the drug classification provided in Table 1 and represent the following: red – anti-inflammatory; green – analgesic; yellow – neurologic; blue – anti-infective; purple – antineoplastic; and gray – miscellaneous. IC_{50} values were determined using a four-parameter variable-slope nonlinear regression and fractionally normalized. Data are presented as means ± SEM from triplicate experiments.
