# Supplementary material for: Discovery of novel antifungal drugs via screening repurposing libraries against Coccidioides posadasii spherule initials
Source: mBio. 2025 Mar 26;16(5):e00205-25. doi: 10.1128/mbio.00205-25 (PMC12077158; doi:10.1128/mbio.00205-25)
Supplement: Fig. S3 — Chemical structures of the top five compounds. [file mbio.00205-25-s0003.pdf]

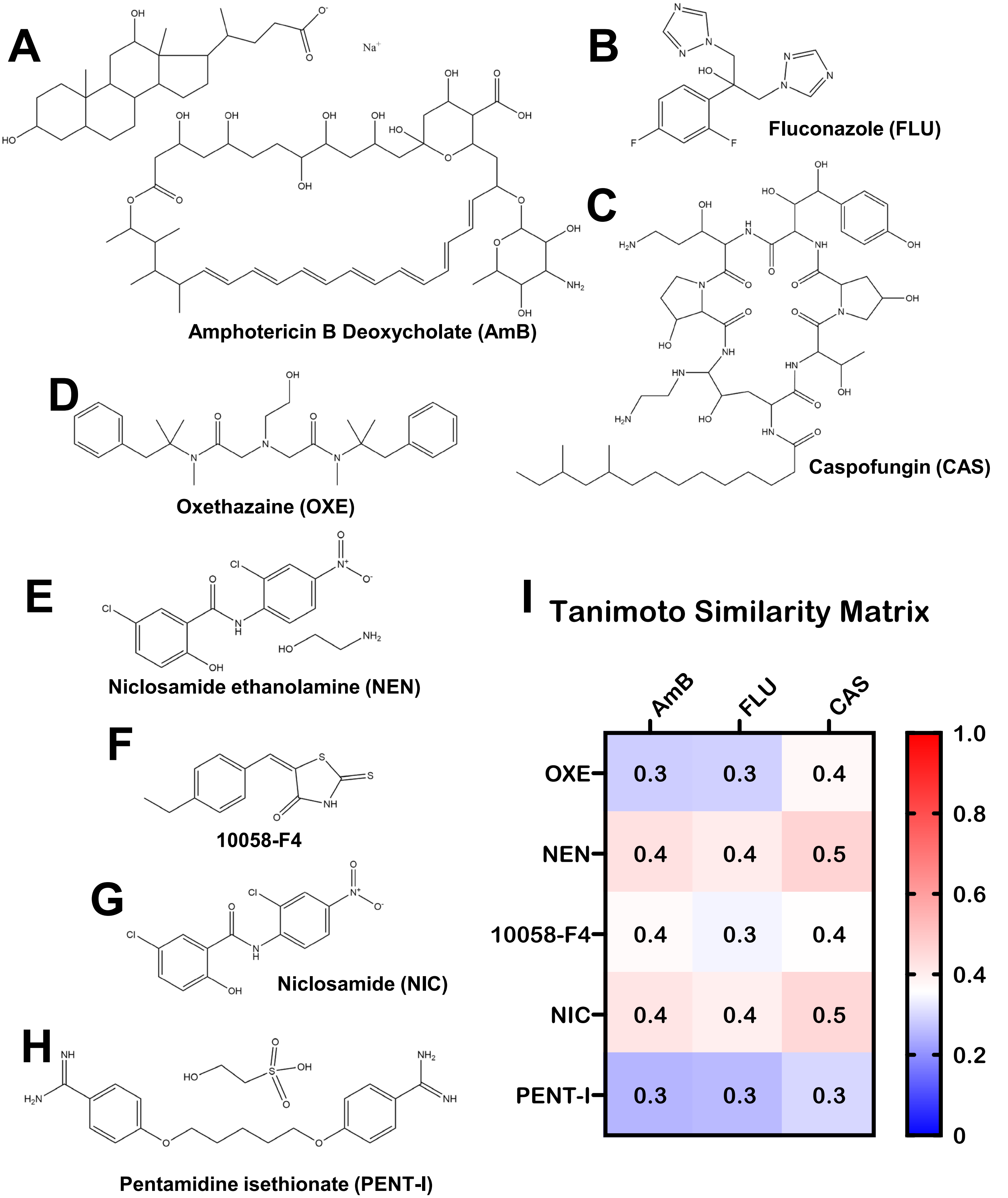

Supplementary Figure 3. Top 5 compounds have unique structures compared to the representative compounds from the Polyene, Azole, and Echinocandin antifungal classes. (A-H) Structures of the tested antifungal agents and the repurposed drugs that show synergy with AmB. **(I)** Tanimoto similarity matrix shows the calculated similarity of each molecule compared to the representative antifungal compounds. Compounds are defined as similar if they have a score ≥ 0.8.
