# Supplementary material for: Discovery of novel antifungal drugs via screening repurposing libraries against Coccidioides posadasii spherule initials
Source: mBio. 2025 Mar 26;16(5):e00205-25. doi: 10.1128/mbio.00205-25 (PMC12077158; doi:10.1128/mbio.00205-25)
Supplement: Fig. S4 — Image flow cytometry gating strategy for profiling Coccidioides posadasii. [file mbio.00205-25-s0004.pdf]

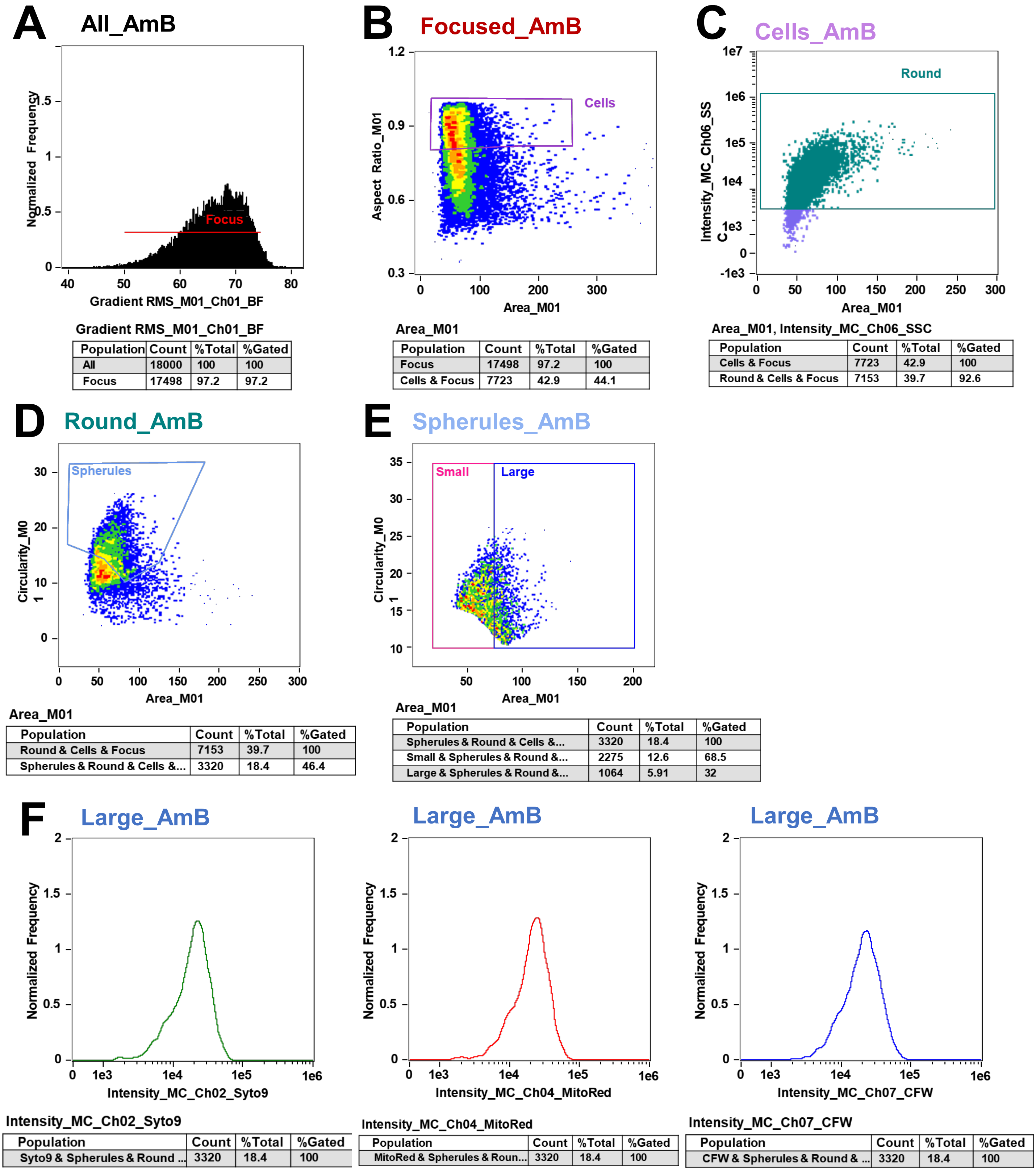

Gradient RMS_M01_Ch01_BF

Population	Count	%Total	%Gated
All	18000	100	100
Focus	17498	97.2	97.2

Area_M01

Population	Count	%Total	%Gated
Focus	17498	97.2	100
Cells & Focus	7723	42.9	44.1

Area_M01, Intensity_MC_Ch06_SSC

Population	Count	%Total	%Gated
Cells & Focus	7723	42.9	100
Round & Cells & Focus	7153	39.7	92.6

Area_M01

Population	Count	%Total	%Gated
Round & Cells & Focus	7153	39.7	100
Spherules & Round & Cells &...	3320	18.4	46.4

Area_M01

Population	Count	%Total	%Gated
Spherules & Round & Cells &...	3320	18.4	100
Small & Spherules & Round &...	2275	12.6	68.5
Large & Spherules & Round &...	1064	5.91	32

Intensity_MC_Ch02_Syto9

Population	Count	%Total	%Gated
Syto9 & Spherules & Round ...	3320	18.4	100

Intensity_MC_Ch04_MitoRed

Population	Count	%Total	%Gated
MitoRed & Spherules & Roun...	3320	18.4	100

Intensity_MC_Ch07_CFW

Population	Count	%Total	%Gated
CFW & Spherules & Round & ...	3320	18.4	100

Supplementary Figure 4. Representative image flow cytometry gating strategy for profiling *Coccidioides posadasii*. (A) Focused cell populations were identified visually. **(B)** Single cells were isolated from aggregates and debris on the basis of area and aspect ratio. **(C)** Gating on area and side-scatter intensity was used to filter out remaining debris and aggregates. **(D)** Spherule initials were separated from arthroconidia based on area and circularity. **(E)** Spherule initials were further separated by size, where cells >75µm were defined as large. **(F)** Representative histograms of the intensities of the three dyes, Syto9, MitoTracker Red CMXRos, and calcofluor white are shown.
